# MiR-31-5p regulates the neuroinflammatory response via TRAF6 in neuropathic pain

**DOI:** 10.1186/s13062-023-00434-1

**Published:** 2024-01-24

**Authors:** Yuqi Liu, Lijuan Wang, Chengcheng Zhou, Yuan Yuan, Bin Fang, Kaimei Lu, Fangxia Xu, Lianhua Chen, Lina Huang

**Affiliations:** Department of Anesthesiology, Shanghai General Hospital, Shanghai Jiao Tong University School of Medicine, Shanghai, 20080 China

**Keywords:** Neuropathic pain, miR-31-5p, TRAF6, Neuroinflammatory response

## Abstract

**Background:**

Neuropathic pain is chronic pain and has few effective control strategies. Studies have demonstrated that microRNAs have functions in neuropathic pain. However, no study has been conducted to demonstrate the role and mechanism of microRNA (miR)-31-5p in neuropathic pain. Accordingly, this study sought to determine the pathological role of miR-31-5p in chronic constriction injury (CCI) -induced neuropathic pain mouse models.

**Methods:**

We used CCI surgery to establish mouse neuropathic pain model. Behavioral tests were performed to evaluate pain sensitivity of mice. Expressions of miR-31-5p and inflammatory cytokines in dorsal root ganglion (DRG) were examined by polymerase chain reaction. Animals or cells were received with/without miR-31-5p mimic or inhibitor to investigate its role in neuropathic pain. The mechanism of miR-31-5p was assayed using western blotting, immunofluorescence staining and dual-luciferase reporter assay.

**Results:**

We found that CCI led to a significant decrease in miR-31-5p levels. Knockout of miR-31-5p and administration of miPEP31 exacerbated pain in C57BL/6 mice. Meanwhile, miR-31-5p overexpression increased the paw withdrawal threshold and latency. TRAF6 is one of the target gene of miR-31-5p, which can trigger a complex inflammatory response. TRAF6 was associated with pain and that reducing the DRG expression of TRAF6 could alleviate pain. In addition, miR-31-5p overexpression inhibited the TRAF6 expression and reduced the neuroinflammatory response.

**Conclusions:**

All the results reveal that miR-31-5p could potentially alleviate pain in CCI mouse models by inhibiting the TRAF6 mediated neuroinflammatory response. MiR-31-5p upregulation is highlighted here as new target for CCI treatment.

**Supplementary Information:**

The online version contains supplementary material available at 10.1186/s13062-023-00434-1.

## Background

Neuropathic pain is a chronic pain characterized by hyperalgesia, allodynia, and spontaneous pain, and the population prevalence of neuropathic pain is estimated to be between 6.9 and 10% [1, 2]. Neuropathic pain can be triggered by many incentive causes, such as diabetes, cancer, postherpetic neuralgia, trigeminal neuralgia, surgery, and trauma [3–5]. Despite many attempts to reveal the etiology of neuropathic pain, its underlying mechanisms remain largely unknown, and its effective control remains inadequate [6]. Consequently, further efforts are urgently needed to identify treatment options for neuropathic pain.

The microRNAs (miRNAs), one of small non-coding RNAs, regulate the expression of messenger RNA by binding to their 3’-untranslated regions [7]. A number of studies have demonstrated that miRNAs are associated with neuropathic pain. For example, 374 differentially expressed circular RNAs were identified in chronic constriction injury (CCI) model rats and negative control rats using circular RNA high-throughput sequencing [8]. Elevated expression of miR-155-5p in the spinal cord contributed to mechanical hypersensitivity via the microglia inflammatory response of microglia in rats with bone cancer pain [9]. Furthermore, Miat promotes nerve injury-induced chronic pain through the miR-362-3p/BAMBI signaling axis pathway [10], and human placental mesenchymal stem cell-derived small extracellular vesicles have alleviated neuropathic pain via miR-26a-5p/Wnt5a in a mouse model of the spared nerve injury [11]. These studies indicated a tight association between miRNAs and neural pain. However, there is no evidence that miR-31-5p affects neuropathic pain.

TRAF6, tumor necrosis factor (TNF) receptor-associated factor, coupling the TNF receptor superfamily to intracellular signaling events, is an important signaling molecule regulating a diverse array of physiological processes, including adaptive immunity, innate immunity, and tissue homeostasis [12]. Recent studies have found a close relationship between TRAF6 and pain. TRAF6 upregulation in spinal cord maintains neuropathic pain induced by spinal nerve ligation (SNL) [13, 14]. TRAF6 inhibited autophagy and increased microglia pyroptosis in complete Freund’s adjuvant (CFA)—induced inflammatory pain [15]. Peli1 facilitated K63-linked ubiquitination of TRAF6 in the ipsilateral spinal cord during CCI [16]. These results indicate the involvement of TRAF6 in the maintenance of neuropathic pain.

Initially, we confirmed the miR-31-5p expression in the dorsal root ganglion (DRG) of CCI mice. We then identified the miR‐31-5p’s function in neuropathic pain. We found a significantly lower expression of miR-31-5p in model mice with chronic sciatic nerve injury compared with controls. MiR-31-5p overexpression relieved neuropathic pain by downregulating the TRAF6-mediated neuroinflammatory response.

## Materials and methods

### Animals

#### CCI animal model

The C57BL/6 wild-type mice were purchased from Shanghai SLAC Laboratory Animal Co in our research. miR31-5p-/- mice were donated by Professor Wang honglin [17]. 6–8 weeks and 15–20 g weight mice were used for experiment.

Sciatic nerve ligation was performed after intraperitoneal injection of pentobarbital. The biceps femoris and superficial gluteus muscle were bluntly dissected using stripping forceps, and three ligatures around the sciatic nerve were performed as previously described [18]. For the sham control operation, the same process was followed without nerve ligation.

In this study, all procedures were carried out in accordance with the Guides For The Care And Use Of Laboratory Animals (National Academy of Sciences, China) and with approval from the Experimental Animal Management Ethics Committee of Shanghai Jiao Tong University School of Medicine (ethical approval number: 2019AW009).

### Behavioral tests

The mice adapted to the environment in the behavior room for 2 days before behavioral tests.

To examine mechanical hyperalgesia, we determined paw withdrawal thresholds using the up-down method. The hind paws were stimulated with a set of logarithmically stiffened von Frey hairs.

To test thermal hypersensitivity, the paw withdrawal latency to a pernicious thermal stimulus was examined using the average of at least three measurement results at 5-min intervals using the Hargreaves instrument. The cut-off time was 20 s to avoid possible hind paw damage.

For detecting cold sensitivity, the paw withdrawal latency was carried out by measuring the time from the rear paw being placed on the plate (0 °C) to the paw flinching from the plate. Three replicates of each assay were performed at 10-min intervals. To avoid tissue damage, 20 s were set as the cut-off time.

Locomotor function, including placing, righting, and grasping reflexes, were evaluated after conducting behavioral tests (Table 1). The following procedures were followed. For the placing reflex, the hind limbs of the mice were held lower than the forelimbs, and the dorsal side of the hind paws contacted the edge of the table. Reflexive hind paw movements on the table were recorded. For the righting reflex, place the animal on a flat surface and record whether it immediately assumes a normal upright posture. For the grasping reflex: when the animal was placed on the wire mesh, record whether the hind paws contact the wire mesh. Each test was repeated five times, with rest interval of 5 min, and the score for each reflex was based on the count of each normal reflex observation.

**Table 1:** Mean changes in locomotor function

Treatment group	Placing	Grasping	Righting
Sham	5(0)	5(0)	5(0)
CCI	5(0)	5(0)	5(0)
miR-31-5p^−/−^	5(0)	5(0)	5(0)
Sham + Scr	5(0)	5(0)	5(0)
Sham + PEP31	5(0)	5(0)	5(0)
Sham + miR-31-5p scramble	5(0)	5(0)	5(0)
Sham + miR-31-5p mimic	5(0)	5(0)	5(0)
CCI + miR-31-5p scramble	5(0)	5(0)	5(0)
CCI + miR-31-5p mimic	5(0)	5(0)	5(0)
Naive + miR-31-5p Scr	5(0)	5(0)	5(0)
Naive + miR-31-5p inhibitor	5(0)	5(0)	5(0)

SEM given in parentheses. n = 8 mice per group; five trials

### Tail vein injection

ScPEP (control peptide) or miPEP31 (50 µg) was intravenously injected into C57BL/6 mice through the tail vein with a 100-μL phosphate-buffered saline volume for the first day. Then we measured the pain behaviors of mice in the next three consecutive days and collected DRG tissues on the third day for the tests later.

### Intrathecal injection

The intrathecal injection method has been previously described [19]. A tail flick indicated correct needle insertion. In brief, the mice were restrained, and a 27-gauge needle was used for injection. All compounds were intrathecally injected between L5 and L6 with a 10-μL volume.

### Cells culture and transfection

Neuron 2a and 293T cell lines were collected in a DMEM medium (Gibco) containing 10% fetal bovine serum (Bioagrio science). Cells were cultured, passaged and were then transfected with Lipofectamine 2000 reagent (Invitrogen). All cells were harvested after transfection for 48 h. MiR-31-5p mimic sequences were forward: 5′- AGGCAAGAUGCUGGCAUAGCUG- 3′, reverse: 5′-CAGCUAUGCCAGCAUCUUGCCU-3′. MiR-31-5p inhibitor sequences were 5’- CAGCUAUGCCAGCAUCUUGCCU-3’.

### Quantitative reverse transcription and polymerase chain reaction (RT‐qPCR)

TRIzol regent (#9109, TAKARA) was used to extract RNA for RT-qPCR, and cDNA was synthesized by reverse transcription of 1 μg RNA (#R323-01-AC, Vazyme). Table 2 lists the primers used.

**Table 2:** Primers for qPCR

Gene	Forward (5′–3′)	Reverse (5′–3′)
GAPDH	AGGTCGGTGTGAACGGATTTG	TGTAGACCATGTAGTTGAGGTCA
TRAF6	TACGATGTGGAGTTTGACCCA	CACTGCTTCCCGTAAAGCCAT
u6	CTCGCTTCGGCAGCACA	AACGCTTCACGAATTTGCGT
miR-31-5p	CTCGGATCCTGTGCATAACTGCCTTCA	CACAAGCTTGAAGTCAGGGCGAGACAGAC
TNF-α	CCCTCACACTCAGATCATCTTCT	GCTACGACGTGGGCTACAG
IL-6	TAGTCCTTCCTACCCCAATTTCC	TTGGTCCTTAGCCACTCCTTC
IL-1β	GCAACTGTTCCTGAACTCAACT	ATCTTTTGGGGTCCGTCAACT

### Western blotting

After pre-washing with phosphate-buffered saline, tissues or cells were lysed and separated using centrifugation. A sodium dodecyl sulfate polyacrylamide gel electrophoresis separation of the lysates was performed, the membranes were transferred to polyvinylidene fluoride membranes, and antibodies were applied. The following antibodies were used: TRAF6 (Abcam, ab40675); GAPDH (CST,2118); H3 (Proteintech, 17,168-1-AP); ERK (CST,4695S); p-ERK(CST,4370S); GFAP (Proteintech, 16,825-1-AP).

### Immunofluorescence and microscopy

OCT was used to embed the tissues from mice. All frozen sections were sliced to slides and frozen slides incubated with antibodies, including mouse anti-calcitonin gene-related peptide (CGRP, Abcam, ab81887), anti-anti-β Tubulin III (TuJ1, abacm, ab78078), isolectin B4 (IB4, Vector laboratories) under a refrigerated environment (4 °C) overnight. After thorough rinsing, the slides were incubated with the respective secondary antibody at 37 °C for 2 h. Samples were imaged under a Nikon E600FN Neurolucida microscope (SP8; Leica), and analyses were carried out manually using ImageJ software.

### DRG microinjection

As described by Zhao et al. [20], sevoflurane was used to anesthetize mice. A midline incision was made in the lower lumbar area. Then, a pneumatic system with a glass micropipette was used to inject TRAF6 short interfering RNA (siRNA; 20 mM, 1–2μL, 5′-AGAAAAGAGUUGUAGUUUU-3′) or scramble miRNA into the exposed and isolated left lumbar facet process at a rate of 50 nL/min. Subsequently, the micropipette was retained for 5–10 min to prevent diffusion before removal and suturing the skin incision. Paralyzed or abnormal mice were excluded from further examinations.

### Luciferase assay

293T cells were cultured on a 12-well plate and co-transfected with miR-31-5p mimic or plasmids containing TRAF6 promoter fragments of human (TRAF6-Luc:5'-CCTCCCTATAGGGCAAGTTGGACTAGGT-3′) using lipo8000 (Beyotime, China). Luciferase activity was measured using the Dual-Luciferase Reporter Assay System Kit (Cat No. DL101-21, Vazyme) after 24 h transfection.

### Statistical analysis

Each animal was randomly assigned to different groups. Results are expressed as mean ± standard deviation. We used GraphPad Prism 8 (GraphPad Software, Inc. La Jolla, USA) for statistical analyses. Analysis of variance and unpaired student’s t-test were used to compare normally distributed data among groups. *P* < 0.05 was considered statistically significant.

## Results

### MiR-31-5p knockout aggravated pain hypersensitivity and activate inflammatory response in mice

**Figure. 1:**
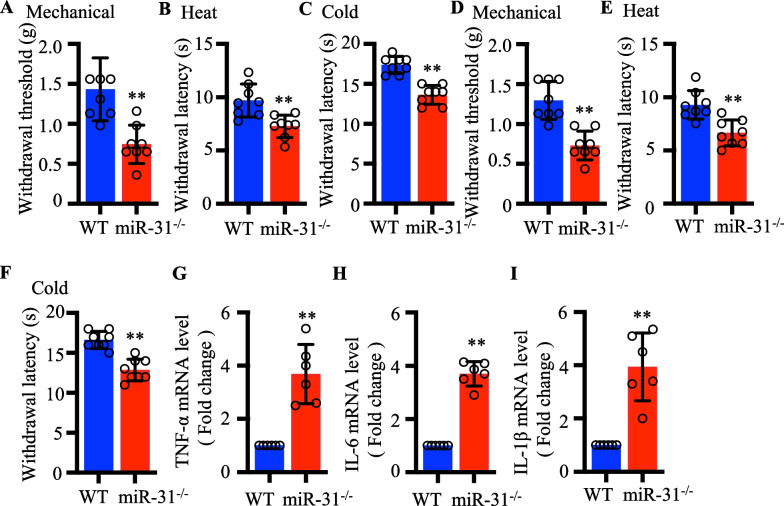
MiR-31-5p knockout aggravated pain hypersensitivity and activate inflammatory response in mice. **a** Mechanical withdrawal threshold in WT and miR-31-5p^−/−^ male mice, ^**^ *P* < 0.01, two- tailed unpaired t-test, n = 8 mice. **b** Heat withdrawal latency in WT and miR-31-5p^−/−^ male mice, ^**^ *P* < 0.01, two- tailed unpaired t-test, n = 8 mice. **c** Cold withdrawal latency in WT and miR-31-5p^−/−^ male mice, ^**^ *P* < 0.01, two- tailed unpaired t-test, n = 8 mice. **d** Mechanical withdrawal threshold in WT and miR-31-5p^−/−^ female mice, ^**^ *P* < 0.01, two- tailed unpaired t-test, n = 8 mice. **e** Heat withdrawal latency in WT and miR-31-5p^−/−^ female mice, ^**^ *P* < 0.01, two- tailed unpaired t-test, n = 8 mice. **f** Cold withdrawal latency in WT and miR-31-5p^−/−^ female mice, ^**^ *P* < 0.01, two- tailed unpaired t-test, n = 8 mice. RT-qPCR analysis of TNF‐α (**g**), IL‐6 (**h**) and IL‐1β (**i**) expression in the DRG of WT and miR-31-5p^−/−^ mice. ^**^P < 0.01, two- tailed unpaired t-test, n = 6 mice.

A large number of studies have shown that miRNAs are related with neuropathic pain. We were given miR-31-5p knockout mice from Honglin Wang Labtory, and on this basis, we were curious about the behavioral differences between miR-31-5p^−/−^ and WT mice. Figure 1a–c illustrates that male miR-31-5p^−/−^ mice exhibited lower pain thresholds than male wild-type (WT) mice in all three tests, including the mechanical pain threshold, heat pain withdrawal latency, and cold pain withdrawal latency tests. The same trend of their behavioral tests was observed in female mice (Fig. 1d–f). Figure 1g–i shows that compared to DRGs of WT mice, miR-31-5p knockout mice had higher levels of inflammatory cytokines including tumor necrosis factor alpha (TNF-α), interleukin 6 (IL-6) and interleukin 1 beta (IL-1β). These results implied that miR-31-5p knockout aggravated pain hypersensitivity and could activate inflammatory responses in mice.

### Mice lacking miR-31-5p exhibited normal innervation patterns and sensory neuron numbers

Next, we collected tissue samples from mice, including the DRG, spinal cord, sciatic nerve, and hind paw cutaneous sensory receptors, exploring the instinct differences between WT and miR-31-5p^−/−^ mice. We used antibodies against β III Tubulin, calcitonin gene-related peptide, and isolectin B4 to label all nerve fibers, fibers from peptidergic nociceptors, and fibers from non-peptidergic nociceptors, respectively. As shown in Additional file 1 & 2, miR-31-5p^−/−^ mice showed no changes in paw peripheral and central innervation patterns, DRG neuron distributions, or sensorimotor behaviors compared with WT mice. These results implied that pain hypersensitivity in miR-31-5p knockout mice was due to sensory neuron-intrinsic miR-31-5p signaling.

### MiPEP31 administration could lead to a decrease in murine pain threshold

**Figure. 2:**
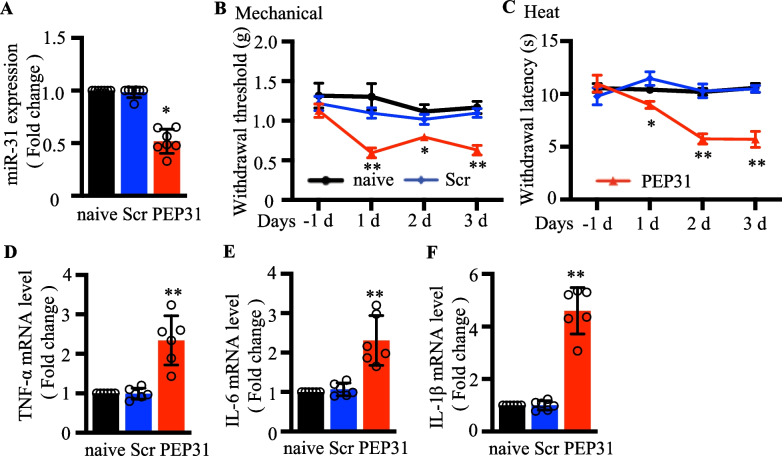
MiPEP31 administration could lead to a decrease in murine pain threshold. **a**–**f** Mice received Scr(scPEP) or PEP31(miPEP31) (50ug in 100 µL phosphate-buffered saline) via tail vein injections. **a** Relative expression of miR-31-5p in the DRG of mice on day 3 after injection. ^*^P < 0.05 vs. naive. One-way ANOVA, n = 7 mice. **b** Mechanical withdrawal threshold in three groups, ^*^ *P *< 0.05, ^**^ *P *< 0.01 vs. naive. two-way ANOVA, n = 8 mice. **c** Heat withdrawal latency in three groups, ^*^P < 0.05, ^**^P < 0.01 vs. naive. Two-way ANOVA, n = 8 mice. RT-qPCR analysis of TNF‐α (**d**), IL‐6 (**e**) and IL‐1β (**f**) expression in the DRG of mice injected with miPEP31after 3 days. ^**^P < 0.01, two- tailed unpaired t-test, n = 6 mice.

The miPEP31 peptide encoded by pri-miRNA-31 acts as a transcriptional repressor, inhibiting miR-31-5p expression [21]. We attempted to downregulate the expression of miR-31-5p by using miPEP31 and examined its impact on behavior of mice. ScPEP (control peptide) or miPEP31 (50 µg) was intravenously injected into C57BL/6 mice through tail vein with a 100-μL volume for the first day. We measured the pain behaviors of mice in the next three consecutive days and collected DRG tissues on the third day. First, a decrease of miR-31-5p in DRG tissue was found in mice treated with miPEP31 (Fig. 2a; *P* < 0.05). During the following 3 days, the miPEP31-treated mice showed mechanical allodynia and thermal hyperalgesia (Fig. 2b, c). Next we further examined the levels of inflammatory cytokines (TNF-α, IL-6, IL-1β) in the DRG and detected higher levels in mice injected with miPEP31 (Fig. 2d–f). These results implied that the absence of miR-31-5p exacerbated pain hypersensitivity.

### Intrathecal injection of miR‐31‐5p inhibitor induced pain hypersensitivities in naive mice

**Figure. 3:**
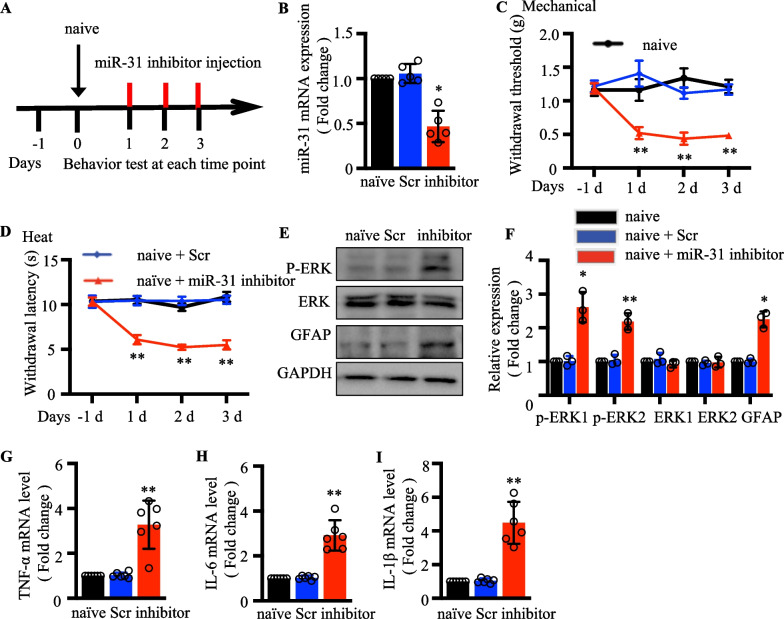
MiR-31-5p inhibitor intrathecal injection induced pain hypersensitivities in naive mice. **a** The description of the animal experimental process. Behavior tests were performed after Scr(scramble) or inhibitor (miR-31-5p inhibitor) administration. **b** Relative expression of miR-31-5p in the DRG of navie mice treated with intrathecal miR-31-5p inhibitors/scrambles was determined by qRT-PCR. ^**^P < 0.01 vs. naive. one-way ANOVA, n = 7 mice. **c** Effects of downregulated miR-31-5p on mechanical allodynia were assessed by withdrawal threshold on post-induction days 1, 2, and 3. ^*^ *P *< 0.05, ^**^ *P *< 0.01 vs. naive; Two-way ANOVA, n = 8 mice. **d** Effects of the downregulated miR-31-5p on thermal hyperalgesia were assessed by heat withdrawal latency at post-induction days 1, 2, and 3. ^*^P < 0.05, ^**^P < 0.01 vs. naïve. Two-way ANOVA, n = 8 mice. **e**–**f** Western blot analysis of P-ERK, ERK and GFAP protein expression in the spinal cord of naive mice at 3 days injected with intrathecal miR-31-5p inhibitor. ^**^P < 0.01 vs. naive; two-way ANOVA, n = 3. RT-qPCR analysis of TNF‐α (**g**), IL‐6 (**h**) and IL‐1β (**i**) expression in the DRG of mice injected with Scr or inhibitor after 3 days. ^**^P < 0.01, two- tailed unpaired t-test, n = 6 mice.

To further clarify the exclusive effect of miR-31-5p of DRG on mouse behavior, we injected miR-31-5p inhibitor intrathecally. A description of the animal experimental process is shown in Fig. 3a. Further clarifying the contribution of miR-31-5p of DRG in pain development, miR-31-5p levels decreased dramatically in naïve mice by injecting an inhibitor intrathecally (*P* < 0.05; Fig. 3b). The functions of miR-31-5p in mechanical pain thresholds and thermal stimulation latencies were observed for three consecutive days. We found that mice treated with the miR-31-5p inhibitors had considerably lower paw withdrawal thresholds to mechanical stimuli than mice treated with scramble miRNA, and similar results were seen for the heat withdrawal latency (Fig. 3c, d), demonstrating that the miR-31-5p inhibitor induced mechanical allodynia and heat hypersensitivity in naïve mice. Central sensitization is associated with the enhanced activity of excitatory neurons, and increased activity of astrocytes in the spinal cord. We examined whether injection of the miR-31-5p inhibitor affected nerve injury-induced central sensitization in the dorsal horn. The protein levels of glial fibrillary acidic protein (GFAP) and phosphorylated and non-phosphorylated extracellular signal-regulated kinase 1/2 (ERK1/2) in the dorsal horn [22, 23] were investigated. Total ERK1/2 levels in L3/4 dorsal horn of spinal cord showed no significant change and spinal GFAP and phosphorylated ERK1/2 (P-ERK1/2) levels were significantly increased in inhibitor-treated mice compared with the naïve and scramble groups (Fig. 3e, f). L4-6 DRGs were also harvested after behavioral tests, and the expression of inflammatory cytokines was measured at mRNA levels. As shown in Fig. 3g–i, mice injected with miR-31-5p inhibitors showed an increase expression of TNF-α, IL-6 and IL-1β. Our results are confirmed the miR‐31‐5p inhibitor induces pain behaviors in naïve mice.

### Downregulated miR‐31-5p levels in the CCI mouse model

**Figure. 4:**
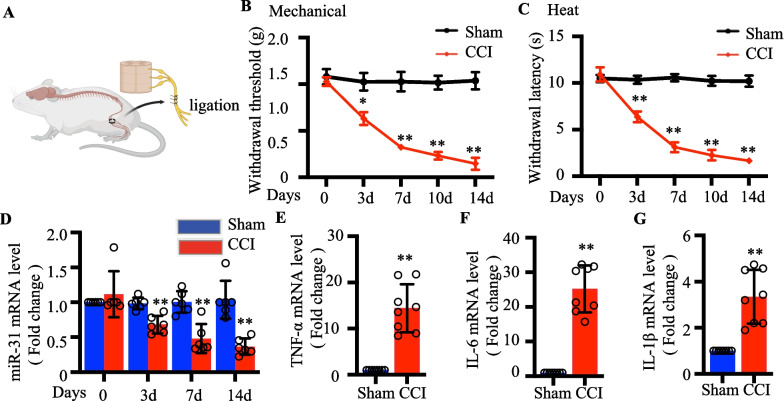
Downregulated miR-31-5p levels in the CCI mouse model. **a** The CCI model was established to induce neuropathic pain in mice. **b** Mechanical withdrawal threshold in sham and CCI mice on postoperative day 0, 3, 7, 10, and 14, ^*^P < 0.05, ^**^P < 0.01 vs. sham. Two-way ANOVA, n = 8 mice. **c** Heat withdrawal latency in sham and CCI mice on postoperative day 0, 3, 7, 10, and 14.^*^P < 0.05, ^**^P < 0.01 vs. sham. Two-way ANOVA, n = 8 mice. **d** RT-qPCR analysis of miR-31-5p expression in the DRG of CCI mice at the different time points, ^*^ *P *< 0.05, ^**^P < 0.01 vs. sham. Two-way ANOVA, n = 6 mice. RT-qPCR analysis of TNF‐α(E), IL‐6(F) and IL‐1β(G) expression in the DRG of sham and CCI mice. ^**^P < 0.01, two- tailed unpaired t-test, n = 6 mice. CCI, chronic constriction injury; ANOVA, analysis of variance; qRT-PCR: quantitative reverse transcription‐polymerase chain reaction; DRG, dorsal root ganglion.

We then established a neuropathic pain mouse model via CCI surgery (Fig. 4a). Consistent with previous results, mechanical allodynia and thermal hyperalgesia were noticed on the ipsilateral paw at 3, 7, 10, and 14 days after CCI (Fig. 4b, c). To find out whether miR-31-5p was abnormally expressed during neuropathic pain progression, mouse ipsilateral L4-6 DRGs were analyzed using RT-qPCR to determine miR-31-5p expression levels. On day 3 after CCI, the miR-31-5p levels were reduced, and this decrease lasted for at least 14 days (*P* < 0.01; Fig. 4d). After CCI, the levels of TNF-α, IL-6 and IL-1β were obviously increased (Fig. 4e–g). These results suggested that miR-31-5p may play a role in the progression of CCI-induced neuropathic pain.

### Intrathecal miR‐31‐5p mimic attenuated neuropathic pain

**Figure. 5:**
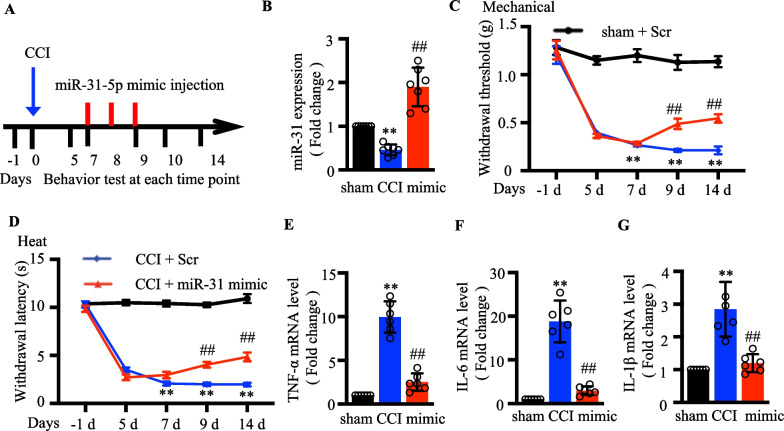
Intrathecal miR-31-5p mimic attenuated neuropathic pain. **a** The description of the animal experimental process. Behavior tests were performed on postoperative days and after scramble or miR-31-5p mimic administration. **b** Relative expression of miR-31-5p in the DRG of CCI mice treated with intrathecal miR-31-5p mimics/scrambles was determined by qRT-PCR. ^**^P < 0.01 vs. sham + scramble; ^##^*P* < 0.01 vs. CCI + scramble. one-way ANOVA, n = 7 mice. **c** Effects of the upregulated miR-31-5p on mechanical allodynia were assessed by withdrawal threshold on post-induction days 5, 7, 9, and 14. ^**^P < 0.01 vs. sham + scramble; ^##^*P* < 0.01 vs. CCI + scramble. Two-way ANOVA, n = 8 mice. **d** Effects of upregulated miR-31-5p on thermal hyperalgesia were assessed by heat withdrawal latency on post-induction days 5, 7, 9, and 14. ^**^ *P *< 0.01 vs. sham + scramble; ^##^*P *< 0.01 vs. CCI + scramble. Two-way ANOVA, n = 8 mice. RT-qPCR analysis of TNF‐α (**e**), IL‐6 (**f**) and IL‐1β (**g**) expression in the DRG of CCI mice injected with Scr or mimic. ^**^P < 0.01, two- tailed unpaired t-test, n = 6 mice.

Based on the above results, we hypothesize that the higher level of miR-31-5p in DRG could alleviate pain in mice. The experimental process for animal experiments is shown in Fig. 5a. MiR-31-5p mimic were injected intrathecally on days 7, 8, and 9 of CCI mice to evaluate the function of miR-31-5p on neuropathic pain. MiR‐31-5p considerably overexpressed in the miR-31-5p mimic injection CCI group compared with the control (*p* < 0.01; Fig. 5b). Figure 5c, d showed that the paw withdrawal threshold decreased significantly after CCI on day 7, indicating that the neuropathic pain model was successfully established. MiR-31-5p mimic injections reversed the reduction in paw withdrawal threshold in CCI group, but this was not seen after the injection of scramble miRNA. The pain withdrawal latency to heat stimuli showed the same pattern as the paw withdrawal threshold. We also collected DRG tissues samples to measure inflammatory cytokines. As presented in Fig. 5e–g, the levels of inflammatory factors in CCI mice were lower after administration of miR-31-5p mimic. These findings elucidated that miR-31-5p exerted a protective function in the emergence of neuropathic pain.

### TRAF6 could relieve neuropathic pain

**Figure. 6:**
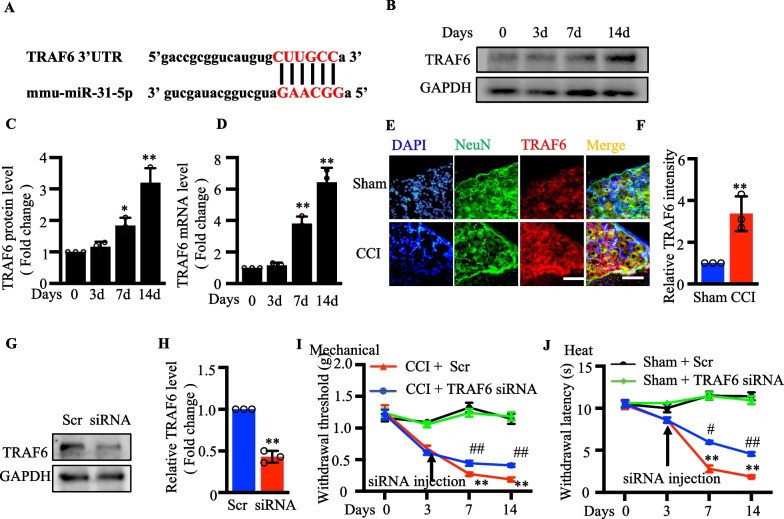
TRAF6 could relieve neuropathic pain **a** The sequence of miR‐31-5p and the position of TRAF6 3′‐UTR. **b**, **c** Western blotting analysis of TRAF6 on days 0, 3, 7 and 14 after CCI surgery. ^*^P < 0.05 vs. sham. ^**^P < 0.01 vs. sham, one -way ANOVA, n = 3. **d** RT-qPCR analysis of TRAF6 on days 0, 3, 7 and 14 after CCI surgery. ^**^P < 0.01 vs. sham, one -way ANOVA, n = 3. **e**, **f** Fluorescence images of TRAF6 (red), NeuN(green) and DAPI (blue) were obtained in DRGs of sham and CCI mice, The scale bar is 50μm, ^**^P < 0.01 vs. sham, Two-tailed unpaired t-test, n = 3. **g**, **h** Western blotting analysis of TRAF6 in the neuron 2a cells with TRAF6 siRNA or scramble transfection. ^**^ *P *< 0.01 vs. scramble. Two- tailed unpaired t-test, n = 3. DRG microinjection of sham and CCI mice received Scr or TRAF6 siRNA. **i** Mechanical withdrawal threshold in four groups, ^**^P < 0.01 vs. sham+ Scr. ^##^*P *< 0.01 vs. CCI+ Scr, two-way ANOVA, n = 8 mice. **j** Heat withdrawal latency in four groups, ^**^P < 0.01 vs. sham+ Scr, ^#^*P *< 0.05 vs. CCI+ Scr, ^##^*P *< 0.01 vs. CCI+ Scr, two-way ANOVA, n = 8 mice.

The TRAF6 target gene was predicted using the Starbase database to determine the downstream targets of miR-31-5p in neuropathic pain. A downstream target gene of miR‐31-5p was TRAF6, with a specific binding site at chr2:101698193–101698198 (Fig. 6a). In addition, we examined TRAF6 expression in the DRGs of CCI model mice. Figure 6b–d shows that TRAF6 expression, including protein and mRNA levels, was significantly increased at days 7 and 14. Meanwhile, we performed a double labeling fluorescence immunohistochemistry assay to evaluate the localization of TRAF6 in the DRG. TRAF6 is mainly expressed in mouse DRG neurons and co-localized with nociceptive neurons (Additional file 1 & 3). Immunohistochemistry revealed an increase in the number of TRAF6-reactive neurons in the CCI group 7 days after the operation compared with the corresponding control sham group (Fig. 6e, f). Then, we transfected Neuro 2a cells with TRAF6 siRNA. After 72 h, TRAF6 protein levels were reduced (*P* < 0.01; Fig. 6g, h). Moreover, we injected TRAF6 siRNA or negative control siRNA into the DRG 3 days after CCI surgery to assess the role of TRAF6 in neuropathic pain. Compared with that of the CCI + negative control siRNA group, the CCI + TRAF6 siRNA group showed significantly blocked development of mechanical allodynia and thermal hyperalgesia (Fig. 6i, j). These findings revealed that TRAF6 was associated with pain and that reducing the DRG expression of TRAF6 could alleviate pain.

### MiR‐31-5p targeted TRAF6 by binding to its 3’-UTR region

**Fig. 7:**
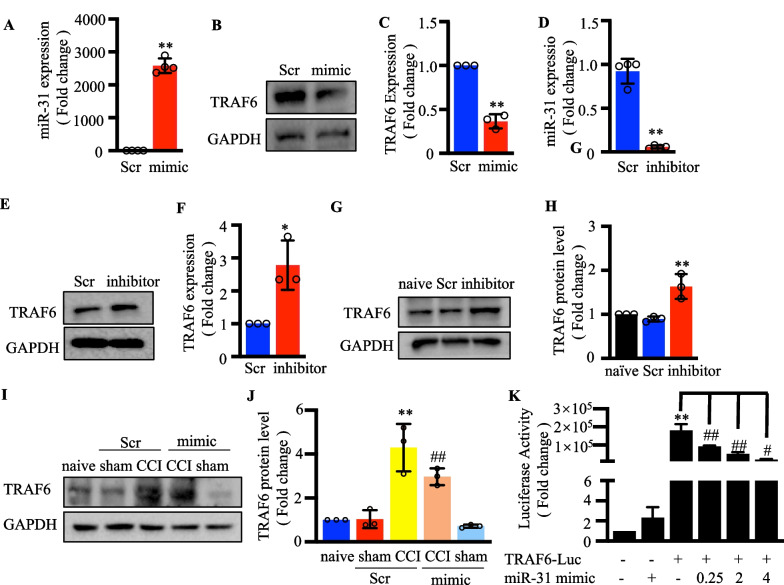
MiR-31-5p targeted TRAF6 by binding to its 3’-UTR region. **a** The relative expression of miR-31-5p with mimic transfection in the neuron 2a cells was determined by qRT-PCR. ^**^ *P* < 0.01 vs. scramble. Two-tailed unpaired t-test, n = 3. **b**, **c** Western blotting analysis of TRAF6 in the neuron 2a cells with miR-31-5p mimic transfection. ^**^ *P* < 0.01 vs. scramble. Two- tailed unpaired t-test, n = 3. **d** Relative expression of miR-31-5p with inhibitor transfection in the neuron 2a cells was determined by qRT-PCR. ^**^ *P* < 0.01 vs. scramble. Two-tailed unpaired t-test, n = 3. **e**, **f** Western blotting analysis of TRAF6 in the neuron 2a cells with miR-31-5p inhibitor transfection. ^*^ *P* < 0.05 vs. scramble. Two- tailed unpaired t-test, n = 3. **g**, **h** Western blotting analysis of TRAF6 protein expression in the DRG of naive mice on day 3 after treated with intrathecal miR-31-5p inhibitors. ^**^P < 0.01 vs. naive; two-way ANOVA, n = 3. **i**, **j** Western blot analysis of TRAF6 protein expression in the DRG of CCI mice at 14 days injected with intrathecal miR-31-5p mimic. ^**^ *P* < 0.01 vs. naive; ^##^ *P* < 0.01 vs. CCI + NS. two-way ANOVA, n = 3. **k** TRAF6 promoter luciferase reporter (TRAF6-Luc) was co-transfected with miR-31-5p mimic in 293T cells for 24 h, and then relative luciferase activity was analyzed. ^**^ *P* < 0.01 vs. naïve, ^#^ *P* < 0.05 vs. previous group, ^# #^*P* < 0.01 vs. previous group. one -way ANOVA

To further demonstrate that miR-31-5p regulates the expression of TRAF6, we transfected Neuro 2a cells with miR-31-5p mimic to upregulate the miR-31-5p level and found that TRAF6 protein expression was inhibited (Fig. 7a–c). Meanwhile, downregulation of miR-31-5p using its inhibitor could increase the levels of TRAF6 (Fig. 7d–f). Next, we tested the DRGs of mice that were treated with miR-31-5p mimic or the inhibitor. Compared with mice injected with scrambled miRNA as a negative control, the miR-31-5p inhibitor increased TRAF6 protein expression in the L4–6 DRGs of CCI model mice (*P* < 0.01; Fig. 7g, h). Moreover, Western blot (Fig. 7i, j) revealed that the miR-31-5p mimic-injected CCI model mice displayed decreased levels of TRAF6 protein. Consistently with the in vivo results, miR-31-5p could negatively regulate the expression of TRAF6.

According to previous studies, miR-31-5p regulates transcription by binding to promoters. In the present study, we constructed a luciferase reporter containing a segment of human TRAF6 promoter to detect the impact of miR-31-5p on promoter activity. Figure 7k showed that the miR-31-5p mimic reduced TRAF6 promoter activity in a dose-dependent manner, indicating that miR-31-5p regulates transcription by binding to the TRAF6 promoter. These results implied that miR-31-5p targets TRAF6 by binding to its 3’-UTR region and that TRAF6 participates in the occurrence of neuropathic pain.

### MiR-31-5p regulated the neuroinflammatory response via TRAF6 in CCI mice models

**Fig. 8:**
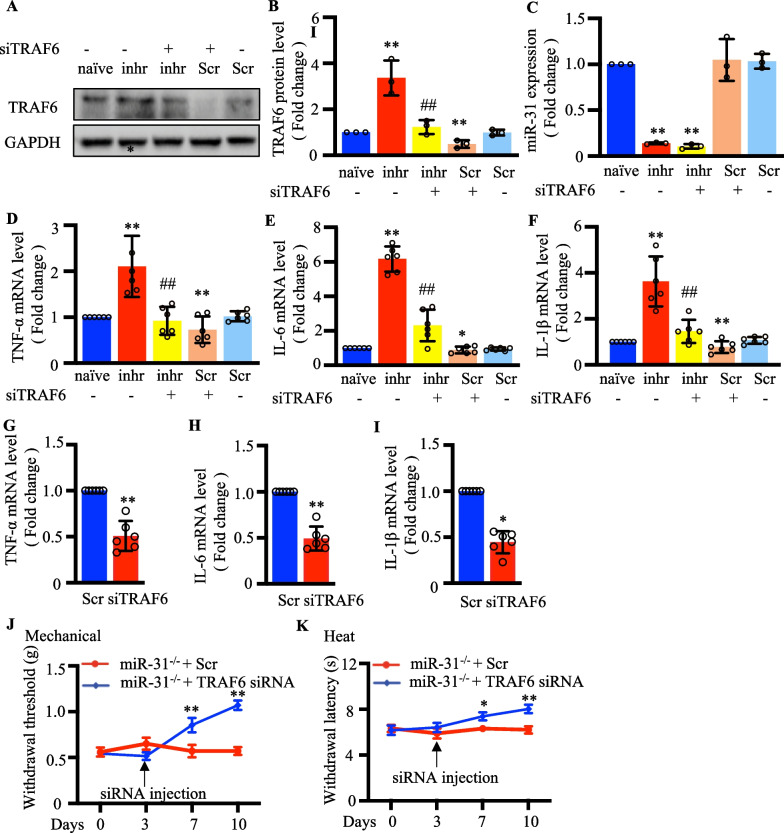
MiR-31-5p regulated the neuroinflammatory response via TRAF6 in CCI mice models. Neuron 2a cells were transfected with inhr (miR-31-5p inhibitor) or Scr(scramble) for 48 h. **a**, **b** Western blotting analysis of TRAF6 protein expression in five groups. ^**^ *P* < 0.01 vs. naive; ^##^ *P* < 0.01 vs. inhr. two-way ANOVA, n = 3. **c** RT-qPCR analysis of miR-31-5p expression in five groups. ^**^ *P* < 0.01 vs. naive; two-way ANOVA, n = 3. RT-qPCR analysis of TNF‐α (**d**), IL‐6 (**e**) and IL‐1β (**f**) expression in five groups.^*^P < 0.05 vs. naive ^**^ *P* < 0.01 vs. naive; ^##^ *P* < 0.01 vs. inhr. two-way ANOVA, n = 6. DRG microinjection of miR-31-5p^−/−^ mice received Scr or TRAF6 siRNA. RT-qPCR analysis of TNF‐α (**g**), IL‐6 (**h**) and IL‐1β (**i**) expression in the DRG of miR-31^−/−^ mice injected with Scr or TRAF6 siRNA. ^*^ *P* < 0.05, ^**^ *P* < 0.01, two- tailed unpaired t-test, n = 6 mice. **j** Mechanical withdrawal threshold in miR-31-5p^−/−^ mice with TRAF6 siRNA, ^**^ *P* < 0.01. two-way ANOVA, n = 8 mice. **k** Heat withdrawal latency in miR-31-5p^−/−^ mice with TRAF6 siRNA, ^*^ *P* < 0.05 vs. miR-31^−/−^ + Scr, ^**^ *P* < 0.01 vs. miR-31^−/−^ + Scr. two-way ANOVA, n = 8 mice

**Fig. 9:**
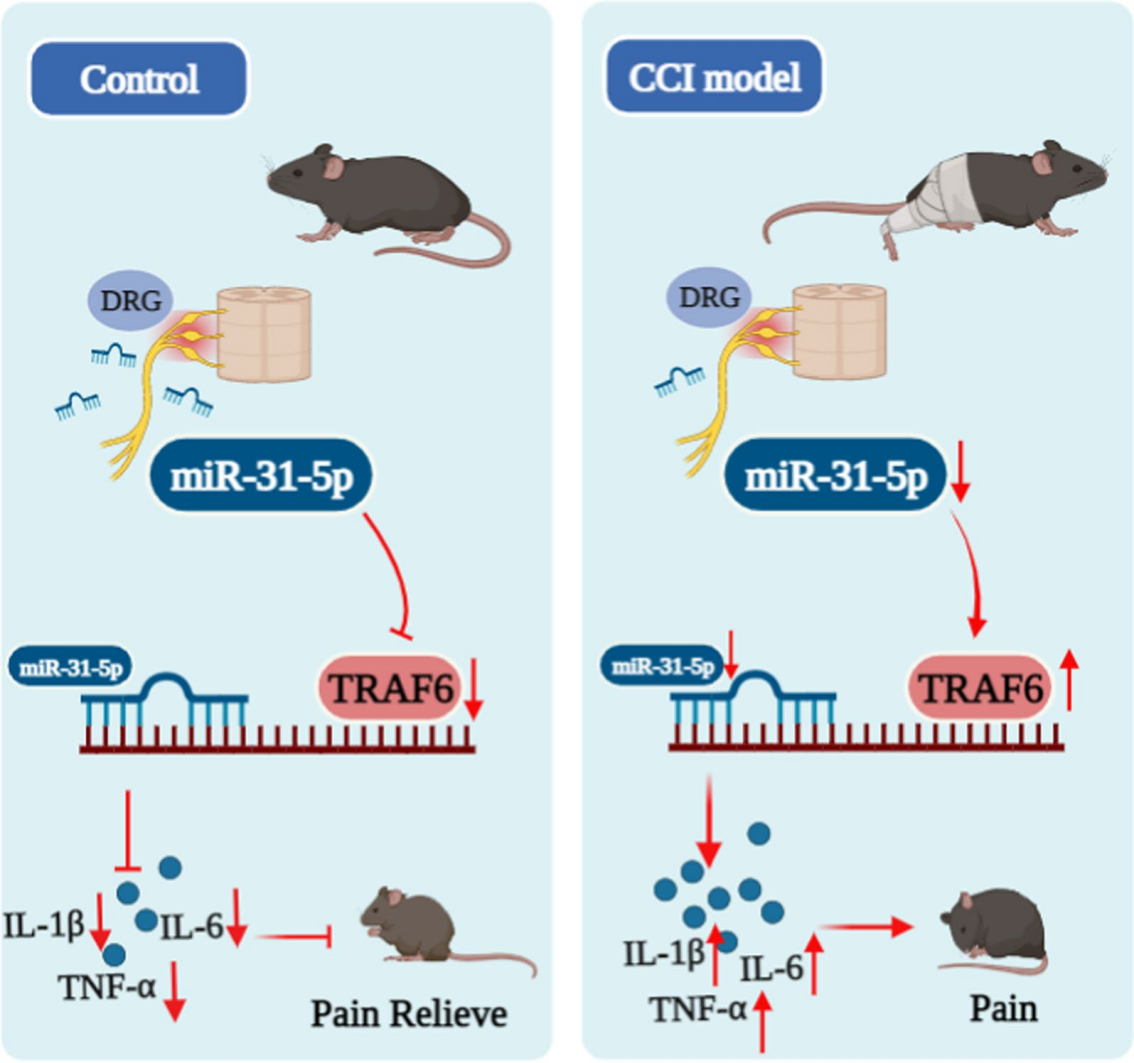
Schematic diagram showing miR-31-5p inhibited TRAF6 transcription and further suppressed the neuroinflammatory response, leading neuroprotective effects against CCI in mice

We transfected neuron-2a cells with miR-31-5p inhibitor and TRAF6 siRNA(siTRAF6). As Fig. 8a, b shows, TRAF6 protein level increased in cells treated with miR-31-5p inhibitor than that in naïve cells or with scrambles siRNA application. At the same time, Neuro 2a cells transfected with the miR-31-5p inhibitor and siTRAF6 showed lower TRAF6 protein levels than those transfected with the inhibitor alone. We also collected cells to test miR-31-5p expression in different groups (Fig. 8c) and found that the miR-31-5p inhibitor lowered miR-31-5p levels, but this was not seen with siTRAF6. These results also suggested that miR-31-5p downregulation could increase the expression of TRAF6, and miR-31-5p is not affected by TRAF6 levels. We continued to test the expression levels of the inflammatory factors including TNF-α, IL-6, and IL-1β, and found that knocking down TRAF6 could reverse the upregulation of inflammatory factors caused by the decrease in miR-31-5p (Fig. 8d–f). Finally, rescue experiments were performed using siTRAF6 microinjection into the DRG of miR-31-5p^−/−^ mice. We examined whether TRAF6 could change the neuroinflammatory response in miR-31-5p^−/−^ mice. Significant decreases in TNF-α, IL-6, and IL-1β levels were observed in miR-31-5p^−/−^ mice after siTRAF6 injection (*P* < 0.05; Fig. 8g–i). After reducing the level of TRAF6 in the DRG, miR-31-5p^−/−^ mice showed increased pain sensitivity thresholds to both mechanical and thermal pain. These results revealed that miR-31-5p regulates the neuroinflammatory response via TRAF6 in a CCI mouse model (Fig. 9).

## Discussion

Neuropathic pain is a kind of chronic pain characterized by spontaneous pain, hyperalgesia, abnormal pain and paresthesia, and it seriously disturbs sleep and causes anxiety and depression, affecting millions of patients worldwide [24]. However, current treatments are often ineffective and inadequate or have serious adverse effects [25]. Particularly, the exact underlying molecular mechanisms of neuropathic pain have not yet been integrally explored. Therefore, new therapeutic strategies and preventative measures for neuropathic pain are crucial.

Studies have shown that several miRNAs are differentially distributed in various areas of the nervous system and contribute to the pathophysiology of pain [26], and some are closely associated with the progression of neuropathic pain by regulating key protein level [27, 28]. For example, miR-3584-5p is overexpressed and can aggravates neuropathic pain by inhibiting Nav1.8 expression through ERK5/CREB pathway [29]. And miRNA-26a is involved in CCI induced neuropathic pain regulated by ciRNA-Kat6 [30]. Of note, Xu et al. identified the levels of several miRNAs in rat serum were changed after SNI surgery through microarray analysis, and suggest that these miRNAs are related to neuropathic pain, including miR-31-5p [26]. Studies have shown that miR-31-5p plays a role in cancer, psoriasis, and diabetic nephropathy [31–33]. Here, our results identify miR-31-5p as a crucial mediator in neuropathic pain that can affect the neuroinflammatory response of DRG tissues in mice. We provide evidence support that miR-31-5p can alleviate neuropathic pain in CCI mouse models by inhibiting the TRAF6 mediated neuroinflammatory response. In addition, use of miRNA31-5p mimic confers protection against CCI model, supporting the therapeutic potential of miRNA-31-5p agonists in the treatment of neuropathic pain.

Neuroinflammatory response has an important role in the progress of neuropathic pain and glial cells, immune cells, and neurons are involved in neuroinflammation [34]. Recently, publications have shown that the innate immune receptors TREM-1 and TREM-2 in microglial are involved in the regulation of pain by modulating neuroinflammatory response [35]. The role of inflammatory factors in neuropathic pain is also found in these research [36]. In particular, Wang et al. have reported that cannabidiol (CBD) can relieve neuropathic pain by inhibiting inflammation factors [37]. Consistent with previous studies, our results showed that the mRNA levels of TNF-α, IL-6 and IL-1β in CCI mice are increased [38]. To further identify the role of miR-31-5p in neuropathic pain, we used miR-31-5p knockout mice, and our results showed that compared with WT mice, both sexes of transgenic mice showed hyperalgesia to mechanical and heat stimuli. Moreover, the miR-31-5p^−/−^ mice showed higher levels of inflammatory cytokines. However, we observed no differences in cutaneous sensory receptors, peripheral, central innervation density, total DRG neuron distribution, and sensorimotor behaviors between miR-31-5p^−/−^ mice and WT mice. Thus, these results suggested that the effects of miR-31-5p in neuropathic pain may due to intrinsic miR-31-5p signaling in sensory neurons.

Pri-miRNA-31-5p encodes a peptide termed miPEP31 that acts as a transcriptional repressor, inhibiting miR-31-5p expression in a sequence-dependent manner [39]. MiPEP31 could promote T_reg_ differentiation to maintain immune homeostasis. Magdalena etc. found that miR-31-5p is strongly involved in the autoimmune process in chronic inflammatory demyelinating polyneuropathy [40]. These studies suggested miR-31-5p may be relevant to inflammation. To verify our hypothesis, we found that miPEP31 increased the levels of inflammatory factors, including TNF‐α, IL‐6 and IL‐1β in DRGs. Consistent with miR-31-5p^−/−^ mice, mice that received miPEP31 presented with mechanical allodynia and thermal hyperalgesia. Meanwhile, intrathecal administration of inhibitor downregulated miR-31-5p levels and contributed to pain behaviors in naïve mice. It has been demonstrated that ERK activation depends on nociceptive activity in spinal dorsal horn neurons [41]. Similarly, in our experiment, we found that GFAP and phosphorylated ERK1/2 levels (markers of astrocyte activation and neuronal activation respectively) increased in the spinal cord of mice subjected to manipulation by miR-31-5p inhibitor. GFAP and P-ERK1/2 expression in mice dorsal horn of spinal cord were significantly up-regulated 3 days after inhibitor injection. Compared with miR-31-5p knockout and miPEP31 injection, intrathecal administration of inhibitors was more specific in downregulating the level of miR-31-5p in DRG. Meanwhile, the miR-31-5p levels were significantly lower in the DRGs of mice with neuropathic pain. For further investigation, we used intrathecal administration of an miR-31-5p mimic, and found that the miR-31-5p mimic could reversed the decrease of miR-31-5p expression in CCI model, relieved pain hypersensitivity and reduced neuroinflammation. Thus, it seems that miR-31-5p may acts as a therapeutic target for neuropathic pain.

To further gain insight into the intrinsic mechanisms of miR-31-5p in modulating pain, we utilized target gene prediction software to screen for target genes. Notably, TRAF6 is one of the downstream molecular of miR-31-5p, has been shown to be involved in multiple signal transduction pathways, mediating adaptive and innate immunity and tissue homeostasis [42]. And recently reported findings revealed that TRAF6 is distributed throughout the nervous system and plays a role in neuropathic pain [14], traumatic brain injuries [43], inflammatory responses in stroke [44], and neurodegenerative diseases [45]. In addition, other studies identified that Sirtuin 1 and reactive oxygen species play a significant role in TRAF6-mediated neuronal damage after stroke, and TRAF6 inhibition displayed neuroprotective effects in ischemic stroke via mitigating oxidative stress and neuroinflammation [44, 46]. These results seemly indicated that TRAF6 is involved in the regulation of neuropathic pain. Further, we investigated the level of TRAF6 in murine CCI model and found that the expression of TRAF6 is highly increased in CCI mice, and the administration of TRAF6 siRNA microinjection to DRGs of CCI mice could relieve neuropathic pain. Therefore, the regulatory mechanism of miR-31-5p in neuropathic pain may exerts through TRAF6. Then, we performed in vitro and in vivo experiments, demonstrated that TRAF6 was negatively regulated by miR-31-5p and the luciferase activity shows that miR-31-5p mimic reduced the promoter activity of TRAF6 in a dose-dependent manner, indicating that miR-31-5p targets TRAF6 by binding to its 3’-UTR regions. In addition, we fully verified the hypothesis through rescue experiments, our results showed that neuroinflammatory response in low-level miR-31-5p can be blocked by TRAF6 siRNA both in vivo and in vitro, indicating that miR-31-5p most likely affects TRAF6 by directly binding to the TRAF6 promoter to inhibit its transcription, thus affecting neuroinflammation and neuropathic pain. Compared with miR-31-5p^−/−^ mice, the presence of miR-31-5p could suppress the activation of TRAF6 and downstream of neuroinflammation signaling pathway, without inducing pain in naïve mice. Therefore, our results confirmed that the miR-31-5p/TRAF6/neuroinflammation signaling pathway is involved in the molecular mechanism of neuropathic pain.

It has been reported that NF-κB acting as a master switch in inflammatory response by regulating excessive levels of IL-6 and TNF- α. NF-κB can increase the expression level of miR-31-5p in intestinal epithelial cells, showed the relationship between NF-κB and miR-31-5p [47]. Moreover, several findings also showed that NF-κB can induce miR-31-5p expression in different disease models [31]. However, Cai et al. showed different results that miR-31-5p can activate MyD88-NF-κB -mediated microglial inflammation in rat subarachnoid hemorrhage model[48]. Other articles showed that the miR-31-5p/GSDMD Axis can regulate pyroptosis and miR-31-5p overexpressing can alleviate the level of TNF‐α, IL‐6 and IL‐1β in sepsis-induced pyroptosis [49]. NF-κB–responsive miR-31-5p led by TNF- α decrease eNOS expression in preeclampsia [50]. And we found similar results in our study. Further analysis these seemingly contradictory results, the common feature of the above articles is that inflammatory factors such as TNF- α act as stimuli to activate the NF-κB pathway and next explore the underlying mechanism. The different experimental conditions may be the reason for the differences. In addition, the limitation of our study is that we did not directly explore the role of NF-κB in miR-31-5p/TRAF6/neuroinflammation signaling pathway, further researches are needed to clarify the mechanism.

## Conclusions

In conclusion, the present study focused on the regulation of miR-31-5p on TRAF6 expression in CCI-induced neuropathic pain. Our findings demonstrated that miR-31-5p inhibited TRAF6 transcription and further suppressed the neuroinflammatory response, leading neuroprotective effects against CCI in mice. These findings suggested that intervening in the miR-31-5p–TRAF6–neuroinflammation pathway may provide preventive strategies for neuropathic pain.

### Supplementary Information

**Additional file 1**. Figure legend of supplementary figures.

**Additional file 2**. Mice lacking miR-31-5p exhibited normal innervation patterns and sensory neuron numbers.

**Additional file 3**. TRAF6 is mainly expressed in mouse DRG neurons.

## Data Availability

The datasets during and/or analyzed during the current study are available from the corresponding author on reasonable request.

## Abbreviations

CCI: Chronic constriction injury
DRG: Dorsal root ganglion
TNF‐α: Tumor necrosis factor alpha
IL‐6: Interleukin 6
IL‐1β.: Interleukin 1 beta
RT-qPCR: Quantitative reverse transcription and polymerase chain reaction
