# Supplementary material for: MiR-31-5p regulates the neuroinflammatory response via TRAF6 in neuropathic pain
Source: Biol Direct. 2024 Jan 24;19:10. doi: 10.1186/s13062-023-00434-1 (PMC10807213; doi:10.1186/s13062-023-00434-1)
Supplement: Supplementary file 1 — Additional file 1. Figure legend of supplementary figures. [file 13062_2023_434_MOESM1_ESM.docx]

Supplementary 1. Mice lacking miR-31-5p exhibited normal innervation patterns and sensory neuron numbers. (A&E) L3-L5 spinal cord segments were collected from WT or miR-31-5p^-/-^ mice and immunostained for IB4, or CGRP with DAPI. The scale bar is 200 μm. (B&F) Hindpaw skin was collected from WT or miR-31-5p^-/-^ mice and immunostained for TuJ1, IB4, or CGRP with DAPI. The scale bar is 100 μm. (C&G) The sciatic nerve was collected from WT or miR-31-5p^-/-^ mice and immunostained for TuJ1 with DAPI. The scale bar is 100 μm. (D&H) Dorsal root ganglion was collected from WT or miR-31-5p^-/-^ mice and immunostained for TuJ1 with DAPI. The scale bar is 50 μm. Two- tailed unpaired t-test, n = 4 mice. TuJ1, anti-β-tubulin III; IB4, isolectin B4; CGRP, calcitonin gene-related peptide; DAPI, 4′,6-diamidino-2-phenylindole.

Supplementary 2. TRAF6 is mainly expressed in mouse DRG neurons. (A) TRAF6 was co-localized with NeuN in DRG neurons. (B) TRAF6 was co-localized with isolectin B4 (IB4) in DRG neurons. (C) TRAF6 was co-localized with neurofilament-200 (NF200) in DRG neurons. (D) TRAF6 was co-localized with calcitonin gene-related peptide (CGRP) in DRG neurons. (E) TRAF6 was not co-expressed with glutamine synthetase (GS). The scale bar is 50μm,
