# Supplementary figures and images for: MiR-31-5p regulates the neuroinflammatory response via TRAF6 in neuropathic pain
Source: Biol Direct. 2024 Jan 24;19:10. doi: 10.1186/s13062-023-00434-1 (PMC10807213; doi:10.1186/s13062-023-00434-1)

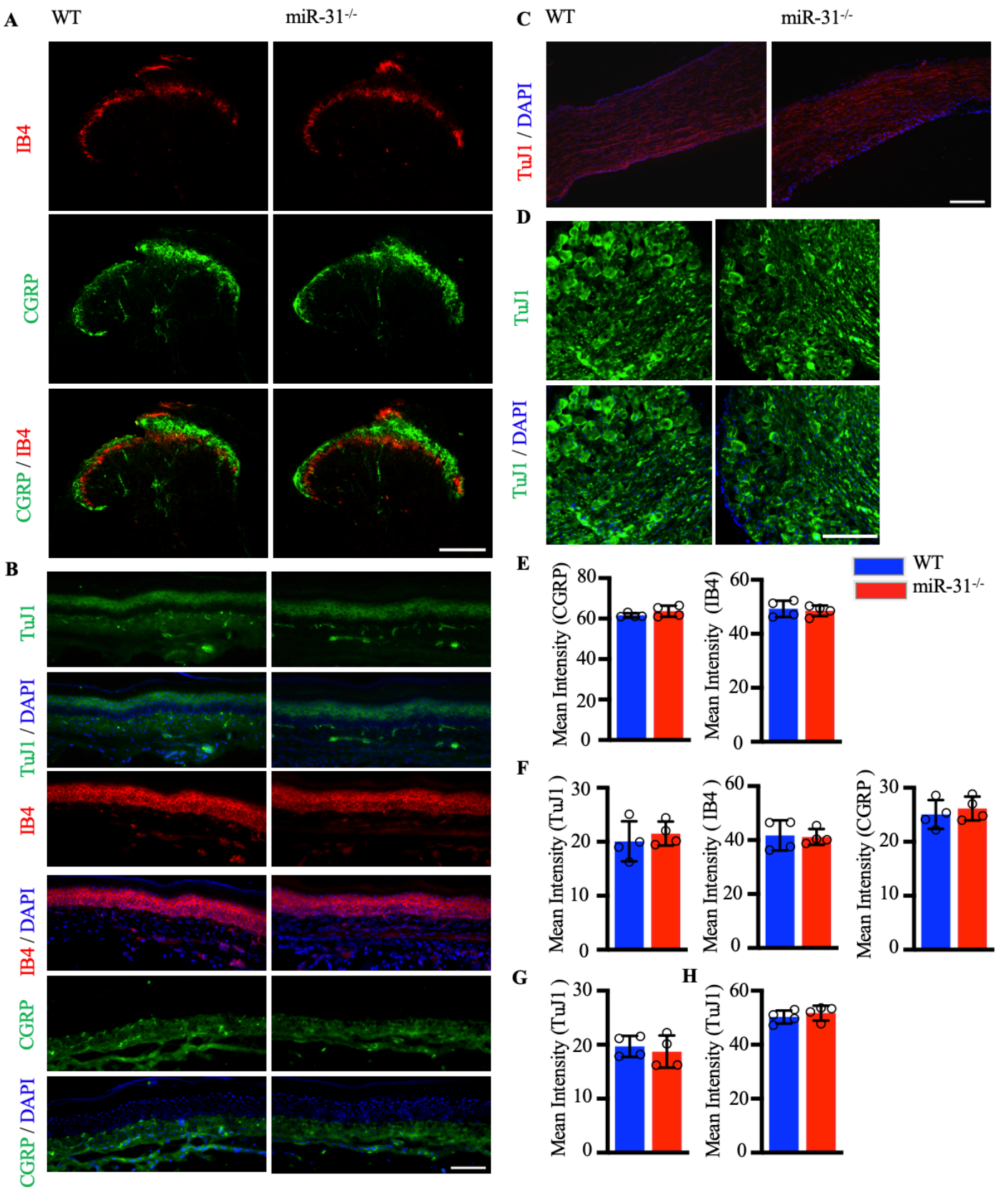

Supplement: Supplementary file 2 — Additional file 2. Mice lacking miR-31-5p exhibited normal innervation patterns and sensory neuron numbers. [file 13062_2023_434_MOESM2_ESM.pdf]

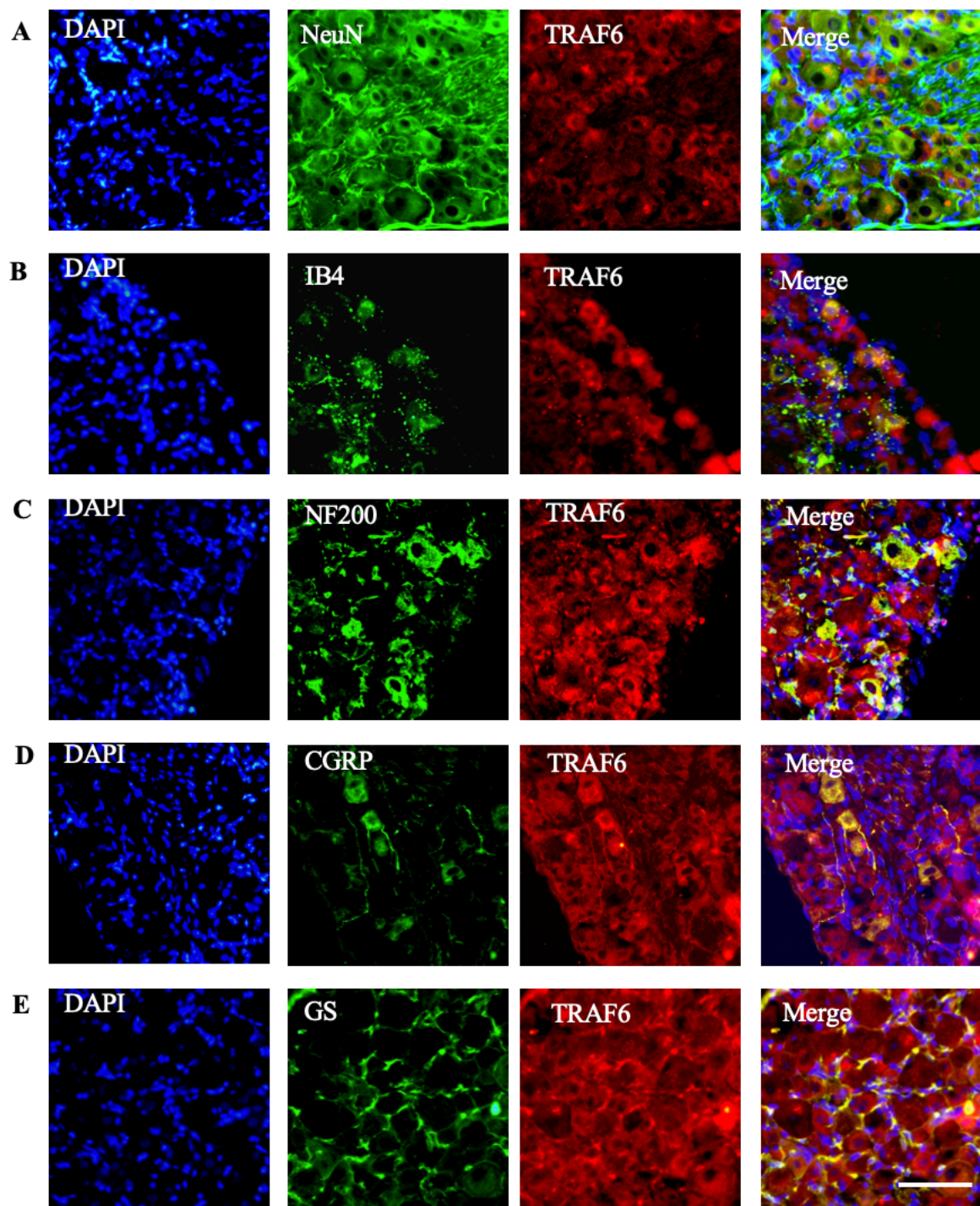

Supplement: Supplementary file 3 — Additional file 3. TRAF6 is mainly expressed in mouse DRG neurons. [file 13062_2023_434_MOESM3_ESM.pdf]
